# Diagnostic Dilemma of a Complex Case of Cerebral Vasculitis: A Rare Probable Drug-Induced Antineutrophil Cytoplasmic Antibody-Associated Vasculitis With Large Vessel Involvement

**DOI:** 10.7759/cureus.61254

**Published:** 2024-05-28

**Authors:** Suria Mano Geran, Sanjiv Bastakoti, Kirsty Levasseur, Zerlene Lim, Shahid Kausar

**Affiliations:** 1 Stroke Medicine, The Dudley Group NHS Foundation Trust, Dudley, GBR; 2 Cardiology, Queen's Hospital, Barking, Havering and Redbridge University Hospitals NHS Trust, London, GBR; 3 Internal Medicine, KIST Medical College & Teaching Hospital, Kathmandu, NPL; 4 Intensive Care Unit, Metrocity Hospital and Research Center, Pokhara, NPL; 5 Rheumatology, Russells Hall Hospital, Dudley, GBR; 6 Radiology, Russells Hall Hospital, Dudley, GBR; 7 Geriatrics and Stroke Medicine, Russells Hall Hospital, Dudley, GBR

**Keywords:** immunosuppressant, stroke, propylthiouracil-induced vasculitis, antithyroid drugs, anca-associated vasculitis

## Abstract

A case of a 43-year-old male with a history of Graves' disease treated with propylthiouracil was investigated for vasculitis and lymphoproliferative disease. However, his clinical picture was complicated by recurrent episodes of neurological symptoms resembling stroke syndrome, which widened the breadth of the diagnostic workup. Extensive investigations, including imaging and biopsies, excluded other possibilities. The patient was treated as probable cerebral vasculitis after identifying new narrowing in the left middle cerebral artery and was treated with pulsed intravenous methylprednisolone, followed by high-dose oral prednisolone and cyclophosphamide. Repeated brain imaging showed further narrowing of the large vessels, which reaffirmed the likelihood of vasculitis necessitating continuation of induction therapy with further maintenance treatment, which led to stabilization of neurological burden and symptom recovery. This case elucidates complexities in reaching the diagnosis of drug-induced antineutrophil cytoplasmic antibody (ANCA)-associated vasculitis, which can present heterogeneously and mimic other clinical entities such as stroke.

## Introduction

Drug-induced vasculitis represents a rare yet significant phenomenon where the inflammation of blood vessels is associated with the administration of pharmaceutical agents. Among the types of vasculitis, John H. Stone classified it as secondary vasculitis, which can be triggered by drugs and is frequently linked with antineutrophil cytoplasmic antibodies (ANCA) [1]. Various drugs, including propylthiouracil (PTU), cocaine, hydralazine, penicillamine, simvastatin, and etanercept, have been implicated in ANCA-associated vasculitis [2]. This condition can affect multiple organ systems, presenting symptoms such as skin rash, arthralgia, renal impairment in the form of crescentic glomerulonephritis, pericardial effusion, and lung involvement.

PTU, an antithyroid medication, is one of the recognized causes of ANCA-associated vasculitis (AAV). Although cross-sectional studies have demonstrated that 15-64% of patients treated with PTU may have circulating ANCA, the progression to ANCA-associated vasculitis is rare [3]. The diagnostic challenge posed by the rarity of drug-induced vasculitis frequently leads to delays in identifying and treating this condition. In this report, we present a case of presumed PTU-induced ANCA-associated vasculitis with multiple clinical manifestations that clinically resembled a stroke, emphasizing the complexity of diagnosis and the critical need for timely intervention.

## Case presentation

A 43-year-old gentleman with an extensive past medical history, including previous intravenous drug use (with a notable cessation of heroin use a decade ago), ongoing methadone therapy, Graves’ disease treated with PTU, a history of lacunar stroke, recurrent uveitis, leg ulcer with venous eczema, and an anxiety disorder, presented with a constellation of systemic symptoms. His presentation was prompted by a few months of feeling unwell, during which a computed tomography (CT) scan of the thorax, abdomen, and pelvis (TAP) revealed widespread lymphadenopathy with hepatosplenomegaly. This prompted an investigative trajectory toward possible systemic vasculitis or lymphoproliferative disease. Biochemical screening for vasculitis returned a positive human leukocyte antigen B27 (HLA-B27), an elevated ANCA-proteinase 3 (PR3) level of 36 (normal level < 2), and a weakly positive lupus anticoagulant, while antinuclear antibody (ANA) and extractable nuclear antigen (ENA), angiotensin-converting enzyme (ACE), syphilis serology, HIV, and hepatitis B and C serology were negative. It is noteworthy that prior to admission, his PTU dosage was increased due to elevated triiodothyronine levels and associated anxiety.

The patient's clinical picture was further complicated by an acute neurological episode characterized by headache, dizziness, blurring of vision, slurred speech, and right-sided limb heaviness. Neurological examination revealed reduced power in the right upper and lower limbs with an upgoing plantar reflex. His medication regimen included clopidogrel, atorvastatin, PTU, mirtazapine, propranolol, and methadone. An initial magnetic resonance imaging (MRI) of the head revealed left cerebral multifocal acute ischemic changes.

**Figure 1:**
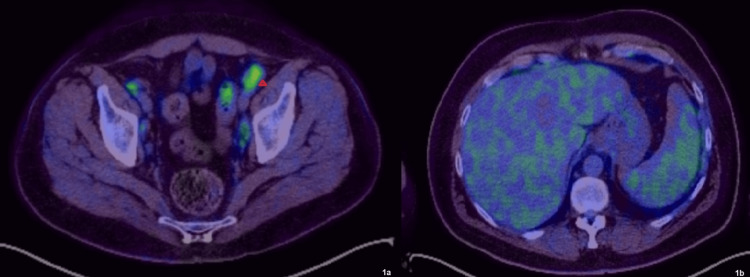
Axial views of FDG-PET/CT. (a) PET/CT demonstrating moderately avid left external iliac lymph node (SUV = 7.0) shown with red arrow. (b) PET/CT demonstrating mild splenic enlargement, which was marginally more active than the liver (SUV max = 4.2 vs. SUV max = 3.7). PET: positron emission tomography; FDG: fludeoxyglucose; CT: computed tomography; SUV: standardized uptake value.

Subsequent investigations encompassed a comprehensive stroke workup, including CT carotid imaging, transthoracic and saline bubble contrast echocardiography, and prolonged Holter monitoring, to exclude thromboembolic events. A whole-body PET-CT scan demonstrated moderately avid lymph nodes and splenic changes without widespread vessel involvement. A biopsy of the most avid lymph node, as seen on the PET-CT (Figure 1), indicated reactive changes and a skin biopsy from the leg ulcer suggested leukocytoclastic vasculitis.

**Figure 2:**
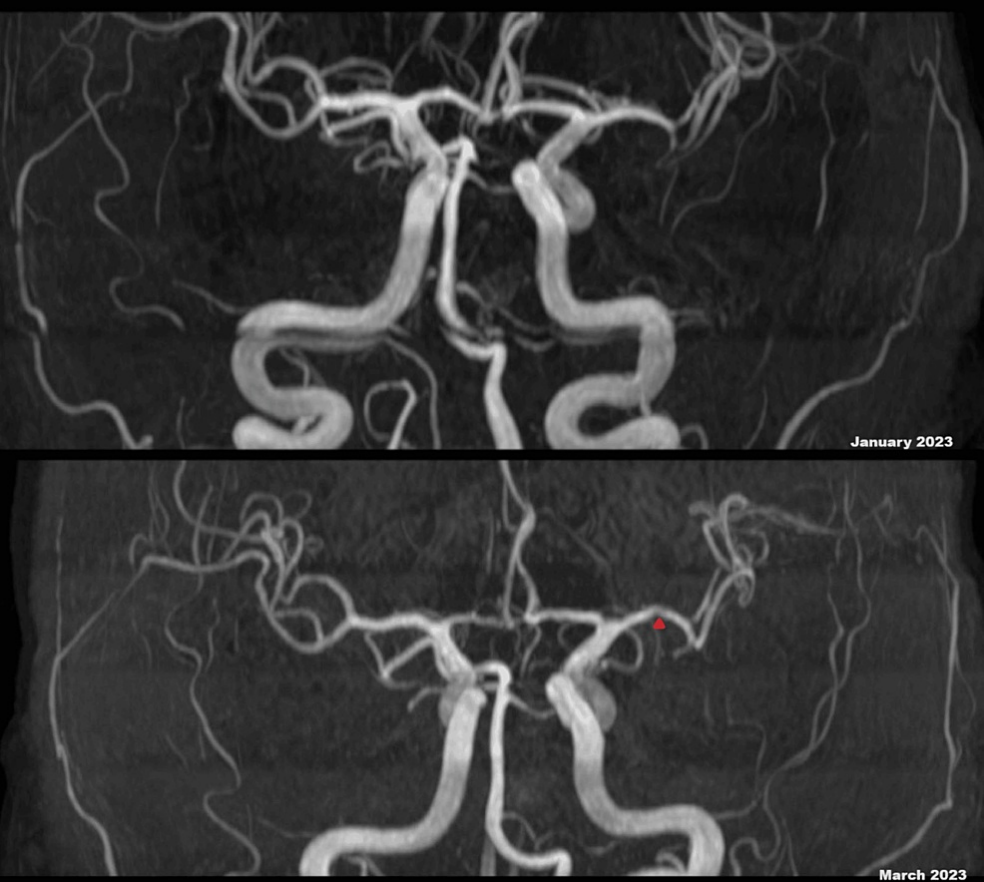
A comparison of MRA of the head done in January 2023 and March 2023. The figure demonstrates the new development of large vessel narrowing, affirming the likelihood of vasculitis. MRA (January 2023) demonstrating left MCA, which appears smooth and of normal caliber. MRA (March 2023) demonstrating irregularity and narrowing of the M1 segment of the left MCA as shown with a red arrow. MCA: middle cerebral artery; MRA: magnetic resonance angiography.

Despite a reduction in ANCA-PR3 levels following the cessation of PTU, repeated MRI scans consistently revealed new multifocal ischemic changes. A repeat magnetic resonance angiogram (MRA) of the head showed irregularity and narrowing of the first segment of the left middle cerebral artery, which was not present on the previous MRA head done two months back (Figure 2). Cerebrospinal fluid (CSF) analysis indicated an elevated protein level consistent with an inflammatory, non-infective process. After multidisciplinary deliberations, and considering the new narrowing observed in the left middle cerebral artery and consistent vasculitis imaging, the decision was made to initiate treatment for cerebral vasculitis. The patient received pulsed intravenous (IV) methylprednisolone, followed by high-dose oral prednisolone, leading to symptomatic improvement.

**Figure 3:**
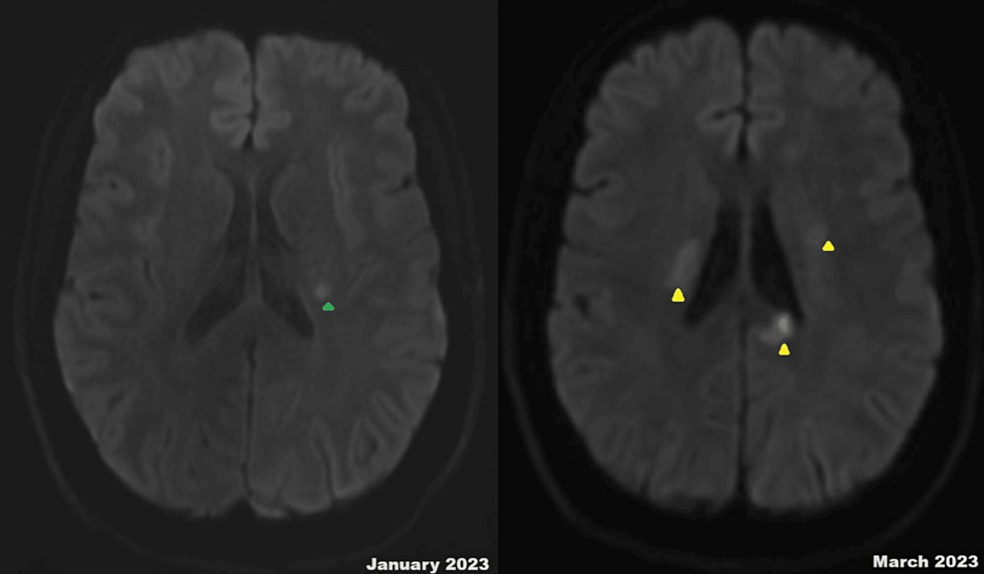
MRI of the head demonstrating the increasing cerebral infarct burden over the course of two months. DWI sequence of the MRI of the head (January 2023) shows a small cerebral infarct in the left corona radiata (green arrow) and left midbrain (not shown). DWI sequence of the MRI of the head (March 2023) shows numerous foci of cerebral infarction (right and left corona radiata, as shown with yellow arrow) as well as bilateral frontal, left temporal, and parietal cortices (not shown). DWI: diffusion-weighted imaging; MRI: magnetic resonance imaging.

Upon discharge, after two months of hospitalization, he was commenced on a planned six-cycle regimen of pulsed IV cyclophosphamide, receiving the first cycle before discharge. However, he was readmitted two weeks later with left-sided weakness and speech difficulties. New MRI imaging indicated additional ischemic foci (Figure 3). The transesophageal echocardiography report revealed a calcified papillary muscle, hence cardiac MRI was done, which ruled out any possibility of apical thrombus and just confirmed incidental calcified papillary muscle. Moreover, a cardiac MRI scan showed a normal aortic root and thoracic aorta. Repeated antiphospholipid screenings were negative.

Therefore, it was decided to continue with vasculitis treatment as planned before. Repeated MRI of the head upon immediate and five-month completion of induction treatment showed no new ischemic changes. In view of the aggressive nature of his presentation and the diagnostic uncertainty, maintenance treatment was initiated with azathioprine, later substituted with mycophenolate mofetil due to gastrointestinal side effects. His neurological function almost recovered back to his baseline, and he remains on a tapering regimen of mycophenolate mofetil, low-dose prednisolone, and antiplatelet therapy. Since the cessation of PTU, his thyroid function has remained stable, and radioiodine therapy is considered for potential future hyperthyroidism. Notably, apart from the skin, no significant organ involvement characteristic of vasculitis was observed throughout his clinical course.

## Discussion

Reflecting upon the case nearly a year after the initial diagnosis of vasculitis, we consider the possibility that this patient's condition was a manifestation of drug-induced ANCA-associated vasculitis (DIV), specifically triggered by PTU. PTU is recognized as one of the more notorious drugs in the etiology of DIV, with a reported median prevalence of 30% [4]. The presentations of DIV can be remarkably heterogeneous, often not paralleling the systemic and acute manifestations typically seen in primary AAV, such as high-grade fever or multi-organ involvement [5].

The diagnostic challenge in this case was compounded by the absence of well-defined criteria for DIV, rendering it a diagnosis of exclusion. The critical initial step upon suspicion of DIV is the discontinuation of the offending drug, which often leads to the resolution of symptoms without the need for further immunosuppressive therapy [6], thereby indicating a better prognosis for DIV compared to primary AAV [7].

In this patient’s case, despite the cessation of PTU, there was a persistence of new cerebrovascular events and an increasing burden of cerebral lesions, suggesting a severe form of the disease. This warranted the use of aggressive immunosuppressive therapy. Interestingly, imaging suggested the involvement of both small and large vessels, a presentation not commonly documented in DIV. This divergence from the typical clinical pattern contributed to significant diagnostic uncertainty and a delay in initiating immunosuppressive therapy.

**Figure 4:**
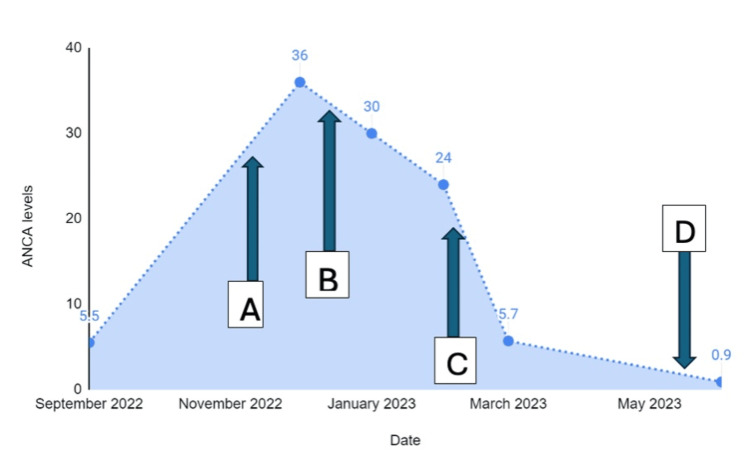
PR3 ANCA level progression in relation to withdrawal of PTU and subsequent treatment with immunosuppressants. (A) PTU dose increment, (B) PTU discontinued, (C) immunosuppression induction, (D) completion of induction therapy. ANCA: antineutrophil cytoplasmic antibodies; PR3: proteinase 3; PTU: propylthiouracil.

The length of treatment for ANCA-associated DIV remains an area of clinical uncertainty. While the removal of the causative drug is paramount, the necessity and duration of maintenance immunosuppression are not well established. The management strategy should be individualized based on the severity of clinical presentation, and the treatment duration is generally shorter compared to primary AAV. In our patient, ANCA levels picked to 36 IU/mL after increment of PTU dose and slowly reduced upon discontinuation of PTU. Subsequently, ANCA levels were completely normalized upon completion of induction treatment with pulsed IV cyclophosphamide. Figure 4 elucidates the trend of PR3 ANCA in relation to the withdrawal of PTU and subsequent treatment with immunosuppressants.

In our patient, the clinical course stabilized sufficiently to warrant a tapering regime of immunosuppression. This case illustrates the nuanced approach required in managing DIV, highlighting the balance between timely drug cessation and the judicious use of immunosuppressive agents in the face of persistent or severe disease.

## Conclusions

This case underscores the complexities inherent in diagnosing and managing DIV, particularly when the presentation deviates from the norm and mimics other serious conditions like cerebrovascular events. Our patient's recovery, following the cessation of PTU and the administration of targeted immunosuppressive therapy, highlights the importance of a high index of suspicion and a personalized treatment approach in cases of suspected DIV.

Further research is warranted to establish robust diagnostic criteria for DIV, which currently remains a diagnosis of exclusion. Longitudinal studies could elucidate the natural history of DIV after the discontinuation of the offending drug, aiding in the determination of the necessity and duration of immunosuppressive treatment. Additionally, the development of biomarkers predictive of DIV could greatly enhance early diagnosis and treatment optimization, potentially improving patient outcomes. The rarity of this condition presents a unique challenge, and collaboration across multiple centers could provide the larger cohorts necessary for more definitive studies.
